# Trends in the Prevalence Gap of Diabetes Diagnosis by City and Province, 2016–2025 - KCHS

**DOI:** 10.56786/PHWR.2026.19.43.5

**Published:** 2026-11-12

**Authors:** Hyo-Jeong Bae

**Affiliations:** Division of Chronic Disease Control, Department of Chronic Disease Prevention and Control, Korea Disease Control and Prevention Agency

Based on the 2025 Korea Community Health Survey (KCHS), the median prevalence of diabetes diagnosis among adults aged ≥30 years across cities, counties, and districts was 9.6%. This nationwide representative value increased by 0.2%p from 9.4% in 2024. The prevalence was highest in Incheon (10.0%) and lowest in Sejong-si (6.4%) (Table 1). The disparity in high-risk drinking prevalence between the highest and lowest regions increased from 2.7%p in 2024 to 3.6%p in 2025 (Figure 1).

## Supplementary Material



## Figures and Tables

**Figure 1:**
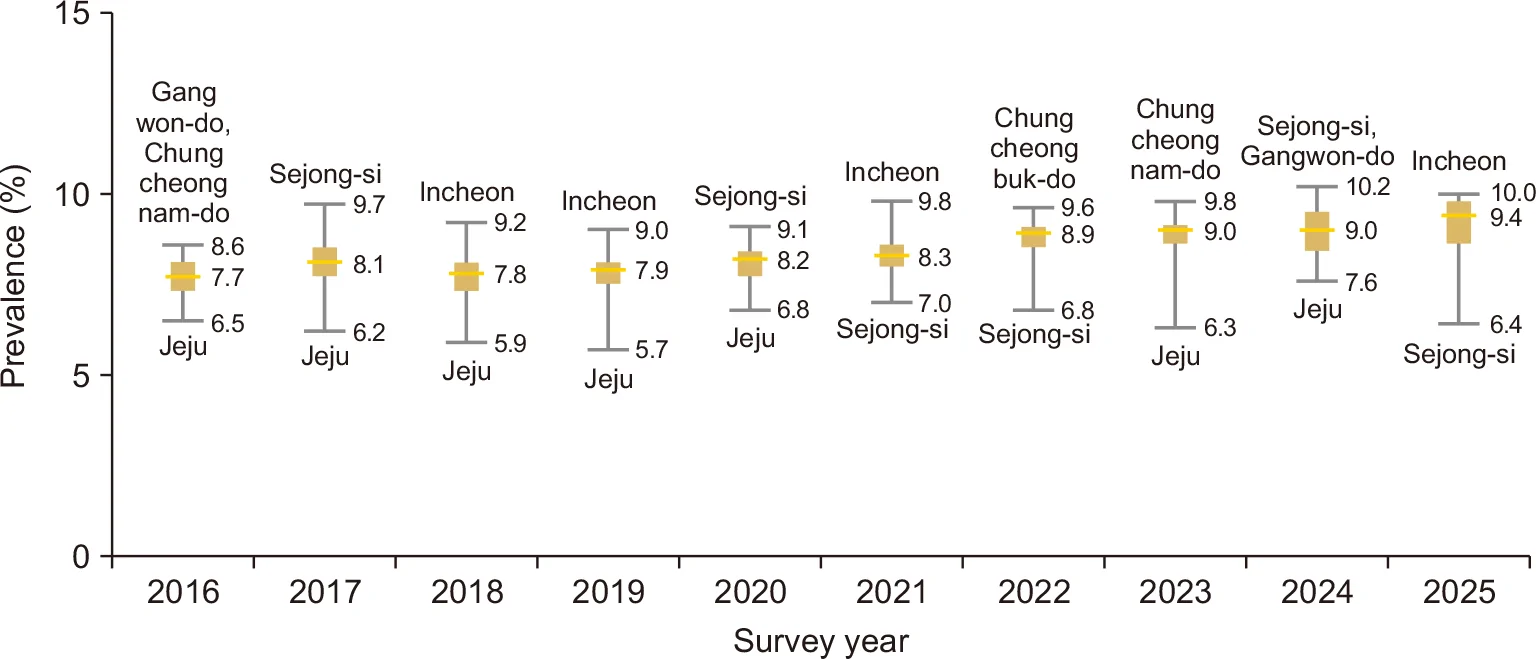
Trends in the prevalence gap of diabetes diagnosis by city and province, 2016–2025 *Prevalence of diabetes diagnosis (aged ≥30 years): the percentage of individuals (aged ≥30 years) who have been diagnosed with diabetes by a physician. **The prevalence gap was calculated as the difference between the maximum and minimum values across the 17 cities and provinces. ※Prevalence rates in Table 1 and Figure 1 were age-standardized using the 2005 projected population.

**Table 1:** Trends in the prevalence of diabetes diagnosis by city and province, 2016–2025

Category	2016	2017	2018	2019	2020	2021	2022	2023	2024	2025
Median	7.8	8.1	7.9	8.0	8.3	8.8	9.1	9.1	9.4	9.6
Seoul	7.6	7.7	7.1	7.5	7.9	7.7	8.5	8.5	8.4	8.5
Busan	7.7	7.4	7.3	7.9	8.2	8.3	8.7	8.7	9.0	9.8
Daegu	7.6	7.8	7.7	7.4	7.7	8.3	9.1	8.6	8.0	8.6
Incheon	7.9	9.1	9.2	9.0	9.0	9.8	9.3	9.7	9.5	10.0
Gwangju	7.1	8.1	7.1	8.0	7.7	8.1	8.8	8.8	9.0	7.9
Daejeon	6.9	8.3	7.8	7.5	7.1	8.0	8.5	9.0	8.9	9.4
Ulsan	8.0	7.7	8.0	8.1	8.0	8.1	8.3	7.3	8.4	8.8
Sejong-si	8.0	9.7	7.4	6.2	9.1	7.0	6.8	9.0	10.2	6.4
Gyeonggi-do	8.1	8.4	8.1	8.0	8.4	8.6	8.9	9.1	9.0	9.4
Gangwon-do	8.6	8.6	8.5	8.4	8.5	9.7	8.9	9.0	10.2	9.5
Chungcheongbuk-do	8.5	8.6	8.1	8.4	8.2	8.9	9.6	9.0	9.3	9.6
Chungcheongnam-do	8.6	8.5	7.8	8.6	8.8	8.9	9.1	9.8	9.5	9.9
Jeollabuk-do	7.3	8.3	7.8	7.9	8.3	8.6	9.1	9.4	8.7	9.4
Jeollanam-do	8.1	8.1	8.1	7.7	7.9	8.6	9.3	9.0	9.5	9.8
Gyeongsangbuk-do	7.6	7.6	8.3	7.9	8.4	8.5	8.9	9.3	9.5	9.8
Gyeongsangnam-do	7.0	7.5	7.3	7.5	7.5	7.9	8.1	8.2	8.2	8.8
Jeju	6.5	6.2	5.9	5.7	6.8	7.3	8.0	6.3	7.6	8.3

Unit: %.

