# Supplementary material for: Trends in the Prevalence Gap of Diabetes Diagnosis by : City and Province, 2016–2025 - KCHS
Source: Public Health Wkly Rep. 2026 Nov 12;19(43):1858–61. doi: 10.56786/PHWR.2026.19.43.5 (PMC13759230; doi:10.56786/PHWR.2026.19.43.5)
Supplement: Translation

# Translation: 시‧도별 당뇨병 진단 경험률 격차 추이, 2016–2025년 – 지역사회건강조사

**Authors:** 효정 배

**Affiliations:** 질병관리청 만성질환관리국 만성질환관리과

2025년 지역사회건강조사 결과, 만 30세 이상 성인의 당뇨병 진단 경험률의 전국 대푯값인 시‧군‧구 중앙값은 9.6%로 전년 9.4% 대비 0.2%p 증가하였다. 시·도별 결과는 인천이 10.0%로 가장 높았고, 세종이 6.4%로 가장 낮았다(표 1). 시‧도 간 격차는 3.6%p로 전년 2.7%p 대비 0.9%p 증가하였다(그림 1).

## Figures and Tables

**그림 1:** 시‧도별 당뇨병 진단 경험률 격차 추이, 2016–2025년 *당뇨병 진단 경험률(30세 이상): 의사에게 당뇨병을 진단받은 30세 이상의 분율 **시‧도 간 격차는 17개 시‧도별 지표값의 최댓값과 최솟값의 차이로 산출 ※표 1과 그림 1의 연도별 지표값은 2005년 추계인구로 연령표준화

**표 1:** 시‧도별 당뇨병 진단 경험률(30세 이상) 추이, 2016–2025년

구분	2016	2017	2018	2019	2020	2021	2022	2023	2024	2025
시‧군‧구중앙값	7.8	8.1	7.9	8.0	8.3	8.8	9.1	9.1	9.4	9.6
서울	7.6	7.7	7.1	7.5	7.9	7.7	8.5	8.5	8.4	8.5
부산	7.7	7.4	7.3	7.9	8.2	8.3	8.7	8.7	9.0	9.8
대구	7.6	7.8	7.7	7.4	7.7	8.3	9.1	8.6	8.0	8.6
인천	7.9	9.1	9.2	9.0	9.0	9.8	9.3	9.7	9.5	10.0
광주	7.1	8.1	7.1	8.0	7.7	8.1	8.8	8.8	9.0	7.9
대전	6.9	8.3	7.8	7.5	7.1	8.0	8.5	9.0	8.9	9.4
울산	8.0	7.7	8.0	8.1	8.0	8.1	8.3	7.3	8.4	8.8
세종	8.0	9.7	7.4	6.2	9.1	7.0	6.8	9.0	10.2	6.4
경기	8.1	8.4	8.1	8.0	8.4	8.6	8.9	9.1	9.0	9.4
강원	8.6	8.6	8.5	8.4	8.5	9.7	8.9	9.0	10.2	9.5
충북	8.5	8.6	8.1	8.4	8.2	8.9	9.6	9.0	9.3	9.6
충남	8.6	8.5	7.8	8.6	8.8	8.9	9.1	9.8	9.5	9.9
전북	7.3	8.3	7.8	7.9	8.3	8.6	9.1	9.4	8.7	9.4
전남	8.1	8.1	8.1	7.7	7.9	8.6	9.3	9.0	9.5	9.8
경북	7.6	7.6	8.3	7.9	8.4	8.5	8.9	9.3	9.5	9.8
경남	7.0	7.5	7.3	7.5	7.5	7.9	8.1	8.2	8.2	8.8
제주	6.5	6.2	5.9	5.7	6.8	7.3	8.0	6.3	7.6	8.3

단위: %.
